# Supplementary material for: Are carbon emissions trading and green financial instruments synergistic? -Comprehensive quantitative research based on content analysis
Source: PLoS One. 2024 Mar 7;19(3):e0298601. doi: 10.1371/journal.pone.0298601 (PMC10919601; doi:10.1371/journal.pone.0298601)
Supplement: S1 Data — (ZIP) [file pone.0298601.s001.zip › Data/地级市Z_F工作报告.zip!/╔╜╬≈/┬└┴║╩╨/└┤╫╘╣1⁄2╓┌║┼í╛┬φ┐╦╩2╛▌═°í┐_2016.docx]

各位代表：

现在，我代表市人民政府向大会报告工作，请予审议，并请市政协委员和其他列席人员提出意见。

2015年工作回顾

刚刚过去的2015年，面对严峻复杂的经济形势和艰巨繁重的改革发展稳定任务，我们在省委、省政府和市委的坚强领导下，在市人大、市政协的监督支持下，团结带领全市人民，深入学习贯彻党的十八大、十八届三中、四中、五中全会和习近平总书记系列重要讲话精神以及省委“五句话”总要求，力同心，攻坚克难，全市经济社会发展在克服困难中奋力前行。

一、强化重点领域投资，经济增长的基本面逐步趋稳。

实施296个省、市重点项目，完成投资963亿元，占全年任务的112.7%。建立政府和社会资本合作（PPP）模式，发布69个总投资930亿元的 PPP 项目，入选国家发改委47个、省级36个，项目数和投资额均位居全省前列。一批重大项目取得实质性进展，离石晋能大土河等5个低热值煤电厂核准开工建设，岚县太钢铁路专用线开通运营，西纵高速临离段和环城高速正式通车，榆横—潍坊特高压吕梁段输电工程开工建设。全市固定资产投资增幅从去年3月份开始由负转正，逐月回升、稳定增长，先后5个月位居全省前三名。坚决执行国家和省减负政策措施，累计为企业减负25.86亿元。扎实开展企业帮扶活动，向驻市金融机构和省金融服务平台推荐融资项目18个；设立企业应急周转保障金5000万元、中小企业发展基金2000万元，为中小企业提供融资担保4.17亿元。多措并举稳定煤炭企业生产，建成8个现代化矿井，努力稳定企业职工队伍，推进煤电联营、煤焦互保，促进了煤炭就地转化。

二、瞄准产业优化升级，新兴产业培育初见成效。

加快煤电一体化发展，全市电力装机容量（含核准在建）达到900万千瓦；加快铝工业发展，氧化铝产量达到1170万吨，全市铝工业发展格局初步形成。加快新能源项目建设，云顶山25万千瓦风电等项目核准开工，中电投岚县河口等3个4.8万千瓦风电和孝义太子科技一期3万千瓦光伏发电项目建成投产。杏花村酒业集中发展区完成投资20亿元，初步形成10万吨白酒生产能力。积极探索军民融合发展，吕梁1号微纳卫星成功发射，云计算中心累计为国家相关核心部门和全国160多家科研院所提供高性能计算服务。旅游标准化景区建设步伐加快，方山北武当和孝义金龙山成功创建国家4A级景区。深入推进农产品流通示范区建设，汾阳山宝食用菌等4个项目建成，交城坤润现代农业产地集配中心等3个项目进入尾声。

三、扎实推进“三农”和扶贫开发工作，农村经济发展保持稳定。

深入落实各项强农惠农政策，建成粮食高产创建示范片27个、高标准农田12.15万亩，实施机械化保护性耕作面积95万亩。大力推进“8+2”农业产业化工程，八大产业实施面积达到55万亩，参与农户扩大到10万户。启动实施沟域经济发展和农业标准化示范市创建工作，创建示范区56个、面积43.8万亩。积极开展农业科技战略合作，与省农科院签订新技术、新品种推广合作项目99个。农村土地承包经营权确权登记颁证工作顺利开展。启动吕梁山片区“三个一”扶贫行动计划，经济林提质增效稳步实施，光伏扶贫进展顺利，护工护理培训按期启动；开展了建档立卡“回头看”，易地扶贫搬迁、金融扶贫、电商扶贫等项目有序推进。全年实现13万贫困人口脱贫。

四、不断加强基础设施，城乡面貌明显改观。

加快市域城镇化布局，新区吕梁大道一期工程建成通车，一期供水和集中供热工程试运行，9座桥梁加快建设，安置区及配套设施建设进展顺利，新区12平方公里框架基本形成。主城区应急水源工程等一批重点项目基本完工，城市供水、供气、供热和污水、垃圾处理等基础设施建设步伐加快，全市城镇化率达到46.24%。深入实施城乡环境整治百日攻坚和人居环境改善工程，城乡面貌明显改善。大力加强路水电气等基础设施建设，国道209省道340中阳过境公路开工建设，新国道太克线方山焦家峪至临县苗家庄、临县梁家会至克虎段升级改造工程进展顺利；中部引黄和配套小水网建设有序推进，龙门供水、千年水库等重点水源工程建设接近尾声，完成水土流失治理面积49.4万亩，8.07万农村人口饮水安全标准得到改善和提高；加大电网投资和改造力度，一批输变电工程建成投产，电网供电质量大幅提升；覆盖勘探区的煤层气管网初步建成，53个加气站开工建设，年输气能力达到25亿立方米，全市气化人口达到134.8万人。

五、大力发展社会事业，民生福祉持续增加。

全年公共财政民生支出达到230.18亿元，占到总支出的84%。实施义务教育“全面改薄”工程，交城、汾阳、离石3个县（市、区）基本达到均衡发展目标。普通高中和县级职教中心标准化建设步伐加快，全市高考二本B类以上达线人数再创新高、突破1.3万人。县级公立医院改革实现全覆盖，120急救中心启动运行，市中心医院、市疾控中心和市妇幼计生服务中心项目奠基开工。城镇职工基本养老保险、城乡居民社会养老保险等各险种参保扩面任务完成，启动城镇职工和居民大病保险工作。开通吕梁到太原城际列车，对市区三分之二的老旧出租车进行了更新换代，实施公交 IC 卡“一卡通”，极大地方便了群众出行。扎实开展脆弱区林业生态综合治理和环境综合整治专项行动，完成造林49.92万亩，推动960个环保重点问题整改，圆满完成6项主要污染物减排任务。严格执行新的《安全生产法》，市县乡村四级安全监管网络进一步完善，安全生产大检查、专项整治和打非治违活动持续开展，全年未发生重大以上事故，部分重点领域保持零事故，全市安全生产形势总体稳定并持续向好发展。

六、深入推进改革开放，政府自身建设得到加强。

试点先行“六权治本”，全面清理行政职权，公布45个市政府部门“两清单、两张图”，并向乡村延伸拓展，为全省提供了可复制、推广的经验和办法。综改2015行动计划圆满完成，统筹推进煤炭、财税、投融资等重点领域改革，出台了金融振兴、科技创新、民营经济发展等一系列政策措施。大力推进招商引资，签约项目108个、签约额 3264.74亿元，签约率全省第一。深入开展学习讨论落实活动和“三严三实”专题教育，持续加强政府系统廉政建设，强化对权力运行的制约和监督，完善议事决策程序，政府工作规范性进一步提高。自觉接受市人大的法律监督、工作监督和市政协的民主监督，主动听取民主党派、工商联和社会各界意见建议，人大代表建议、政协委员提案办结率均达到100％。与此同时，工会、团委、妇联等人民团体作用有效发挥，国防动员、人民防空、民兵预备役建设进一步加强；粮食供销、外事侨务、文物保护、防震减灾、民族宗教、档案方志、应急管理、对台、气象等工作取得新的进步。

各位代表，回顾2015年工作，我们积极应对挑战，经受了严峻考验，全年完成地区生产总值955.8亿元，同比下降4.7%；规模以上工业增加值516.1亿元，下降11.9%；公共财政预算收入90.6亿元，下降30.57%；固定资产投资1166.4亿元，增长14.7%；社会消费品零售总额406亿元，增长4.4%。城镇居民人均可支配收入22903元，增长6.6%；农村居民人均可支配收入7193元，增长6.5%。各项约束性指标圆满完成。这些成绩的取得来之不易，是市委统揽全局、科学决策的结果，是各级各部门团结协作、顽强拼搏的结果，是全市人民同心同德、共同奋斗的结果。在此，我代表市人民政府，向各位人大代表、政协委员，向离退休老同志，向各民主党派、人民团体和各界人士，向驻吕解放军、武警官兵和公安干警，向全市人民及所有关心支持吕梁发展的朋友，表示衷心的感谢，并致以崇高的敬意！

看到成绩的同时，我们也要直面问题。一是地区生产总值、规模以上工业增加值、一般公共预算收入三项主要经济指标大幅下降，经济下行压力持续加大，稳增长的任务异常艰巨。二是煤炭经济占到全市工业经济的48.6%，“一煤独大”的结构没有发生根本改变，传统产业改造升级步伐缓慢，新兴产业和接续产业尚未形成规模，加快培育新的增长动力刻不容缓。三是农业生产方式粗放，抵御自然灾害能力不强，粮食产量同比减少42.58%；主要农产品价格下跌，特别是红枣、玉米等农产品出现严重滞销，直接影响了农民收入的稳定增长。四是全市贫困人口还有48万，占到全省总数的五分之一、全市总人口的八分之一，而且因灾、因病、因学、因婚返贫现象时有发生，脱贫攻坚任务十分艰巨。五是路、水、电等基础设施比较薄弱，教育、卫生、社保等民生事业欠账较多，一些事关群众切身利益的问题尚未有效解决。六是部分工作人员精神状态不佳，不敢为、不愿为、不会为、不作为等现象仍然存在，政府职能转变、简政放权还不到位，政府建设、干部作风与人民群众期盼还有差距。对此，我们一定要清醒认识、高度重视，勇于担当、认真解决，决不辜负人民重托。

2016年工作安排

2016年是“十三五”开局之年，是坚决打赢脱贫攻坚战、全面建成小康社会的关键之年。我们要全面贯彻党的十八大和十八届三中、四中、五中全会及省委十届七次全会精神，深入落实习近平总书记系列重要讲话精神，按照“五位一体”总体布局和“四个全面”战略布局，坚持发展是第一要务，牢固树立“五个发展”新理念，按照省委“五句话”总要求，牢牢把握市委“新布局、新发展、新形象”总体要求，积极适应经济发展新常态，坚持改革开放，坚持稳中求进工作总基调，坚持稳增长、调结构、防风险，以脱贫攻坚统揽经济社会发展全局，着力加强供给侧结构性改革，去产能、去库存、去杠杆、降成本、补短板，突出产业支撑，强化投资拉动，创新发展动力，保障改善民生，积极主动作为，统筹推进创新发展、协调发展、绿色发展、开放发展、共享发展、廉洁和安全发展，保持经济平稳健康发展，努力实现“十三五”时期全市经济社会发展的良好开局。

综合考虑各方面因素，经过认真测算，预计全年地区生产总值增长3%；固定资产投资增长15%，社会消费品零售总额增长6%；公共财政预算收入增长2.5%；城镇居民人均可支配收入和农民人均可支配收入分别增长6.5%和6.5%以上。万元GDP 能耗，万元 GDP 二氧化碳排放量，万元 GDP 用水量降幅，二氧化硫、化学需氧量、氨氮、氮氧化物、粉尘、烟尘等减排指标，按要求完成省定任务。这些主要指标体系的设定，考虑了我市实际，结合了省考核目标，兼顾了当前与长远、体现了需要与可能。尽管我市面临的经济下行压力持续加大，发展中还存在很多困难和问题，但经济增长有一定基础，积极因素正在不断累积。只要我们坚定信心，保持定力，充分调动全市上下的积极性和创造性，以爬坡过坎的拼劲、抓铁有痕的韧劲，自加压力，奋力赶超，就一定能克服困难，走出困境。

今年要重点抓好以下八方面工作：

一、深化结构性改革，着力提高发展的质量和效益

把供给侧结构性改革作为经济发展的强大动力，优化产业结构调整，加快产品提档升级，淘汰一批落后产能，整合一批过剩行业，扶持一批龙头企业，培育一批新兴产业，用改革的减法改造传统产业，用改革的加法培育新的经济增长点。

千方百计稳定煤、焦、钢等主导产业基本面。坚决落实省政府和我市各项税费减免政策，清理规范涉企收费，减轻企业负担。继续坚持市、县领导干部联系帮扶重点企业制度，完善“一企一策、分类指导”帮扶措施，推动煤电联营、煤电铝联营、煤化联营、煤焦钢联营，帮助企业解决实际困难和问题。多措并举化解煤、焦、钢等过剩产能，制定并实施推动产业重组、处置“僵尸企业”的方案和分类推进办法，摸清底数、建立台账。积极引导企业完善内部管理制度，千方百计降耗增效；同时，按照市场化要求，合理配置生产经营要素，继续推行资源整合和企业兼并重组，通过股权置换、采矿权转让、资本整合等多种方式，组建一批市场竞争能力和抵御风险能力较强的大型企业集团，推动煤、焦、钢等行业脱困转型。煤炭产业要严格落实“五个一批”，从严控制煤炭资源配置和煤矿项目审批，精心组织企业生产，规范煤矿建设和生产秩序，建立煤炭监管信息平台，全面提高煤矿标准化生产水平。

焦化产业要重点支持孝义新型煤化工等焦化园区延伸产业链条，抓好交城润锦化工、孝义金岩等一批现代煤化工项目，推动以焦为主向焦化并举、以化为主转变。钢铁产业要由普通钢为主向优质钢、特种钢、钢制品发展，加大技术改造力度，拉长延伸产业链；稳妥推进中钢、文水海威等企业升级，提高终端产品和高附加值产品比重。

不遗余力培育壮大新兴产业。以电力体制综合改革为突破口，优化资源配置，降低用电成本，重点引进一批现代高载能企业，积极探索煤电铝材一体化、战略性新兴产业齐驱并进的新路径。抓好电力产业这个引擎，充分发挥总装机900万千瓦的电力优势，利用我市目前日益完备的电网，在有效降低工业项目电力成本的基础上，主动加强与国内外一流的高载能企业对接联系，引进一批以华为为代表的高科技和以中铝、信发为代表的铝材加工等高载能项目。加快建设离石晋能大土河等5个低热值煤发电项目，确保文水国金二期、离石晋能大土河两个电厂年底如期建成投产，同步实现市区集中供热。加快中铝华润2×66万千瓦自备电厂核准步伐，推动临县等4个2×100万千瓦坑口电厂项目开展前期工作。加快规划布局工业园区电网，重点抓好交城、文水、孝义三个省级开发区建设，进一步完善开发区功能，突出抓好开发区内电网配套；统筹推进其他工业园区电网规划和建设，尽快形成市域大电网与园区小电网互联互通的电网格局。抓好铝工业这个重点，充分发挥电力、电网、电改优势和占全省近一半、全国六分之一的铝土矿资源优势，进一步优化铝工业布局，重点建设兴县、交口、孝义、中阳和柳林现代铝产业园区，推进煤电铝材一体化发展。要以中铝华润50万吨轻合金项目为重点，加快建设吕梁百万吨级铝循环基地，积极推动交口信发、孝义兴安化工等铝系产业项目尽快落地，建设全国重要的铝工业基地。抓好白酒产业这个优势，依托汾酒集团，加快完善杏花村酒业集中发展区，着力延伸白酒产业链条，推动白酒研发、生产、贮存、包装、物流配送一体化发展；进一步强化汾阳、文水等区域化名酒产地理念，突出抓好中汾酒城规模化生产，启动开展地方品牌争创国家驰名商标、中国名牌产品活动，重点扶持创建一批地方名白酒品牌，构筑国有与地方品牌互补、产品错位发展的格局；大力推广“原酒质押”、“白酒商标质押”等融资模式，以企业为主体设立白酒储藏基金，年内争取建成国家级白酒质量检验检测中心，适时筹备召开国家级清香型白酒博览会、交易会和酒业发展论坛等活动，不断提升我市全国最大清香型白酒基地的影响力和竞争力。抓好新能源、新材料产业这个亮点，围绕建设清洁能源基地，积极推动晋能集团利用四荒地、华润集团利用交口露天煤矿复垦地建设大型光伏电站，开工建设孝义巨隆等4个总装机6.6万千瓦光伏电站，建成孝义太子光伏电站；加快建设华电汾阳10万千瓦、云顶山25万千瓦风电项目，新开工国电龙源岚县界河口等4个总装机25万千瓦风电项目，争取岚县河口等3个风电项目上半年并网，建成方山马坊等2个风电项目。推进地面煤层气开采和井下瓦斯抽采，逐步完善1300公里煤层气管网建设，年内争取新区正式通气。重点推进文水晋能太阳能电池及组件、岚县江川国威软磁芯二期及交城利虎玻璃等一批新材料项目加快建设。

挖掘潜力实现旅游服务业提质增效。推进旅游业提档升级，全面抓好以国省干道、高速公路、飞机场、火车站为重点的旅游宣传工作，塑造吕梁旅游新形象。深入挖掘红色文化、廉政文化、黄河黄土风情和自然风光等旅游资源，加快标准化景区建设，重点打造兴县晋绥革命纪念馆、方山于成龙廉政文化园、临县碛口古镇、汾阳中汾酒城、孝义梦幻海水上乐园等特色景区，启动“交城玄中寺、卦山、吕梁英雄广场”国家5A级景区创建工作，大力开发农家乐、休闲农庄、乡村客栈、农业观光园等乡村旅游项目。着力完善旅游公路、旅游厕所和停车场等基础设施。组织策划旅游招商等系列活动，引进省内外知名企业开发我市旅游资源，加快融入太原、晋中旅游经济圈，打造吕梁特色旅游品牌。加速电子商务与传统产业深度融合，全面实施“互联网+流通”行动计划，引导全市批发、零售、商贸服务等行业深化互联网应用。利用淘宝大学等知名电商机构对各级领导干部进行专业化、系统化培训。积极对接引进阿里巴巴、京东商城等知名电商，争取淘宝“特色中国·吕梁馆”上线运营；重点支持石楼树德枣业等10户企业申报省、市级电商示范企业，支持孝义高新科技产业园等3户园区申报省、市级电子商务示范基地；推进农村电子商务全覆盖，力争建设13个县级电商综合服务中心、100个乡镇物流中心、1000个村级电商便民服务网点。支持航空物流业快速发展，积极发展临空经济。规范第三方物流发展，推广统一配送、共同配送等模式，着力解决物流配送“最后一公里”和农产品输出难、输出贵的问题。加快专业市场建设，积极整合行业和产业规模，建设一批地方美食、瓜果蔬菜、数码家电等各类生产生活资料专业街、专业市场，大力培育养老、家政、健康、信息等新的消费增长点，持续做大消费总量。

全力以赴扩大重点领域投资。充分发挥投资在促进经济增长中的关键作用，多方位开辟投资渠道、多形式挖掘投资潜力、多领域优化投资结构，力争全年固定资产投资完成1341亿元。深入开展“项目创新年”活动，“六位一体”统筹推进重点工程建设。围绕重大基础设施、产业转型、城镇化和生态环保、民生社会事业建设等四个方面，推进十大领域、624个重点工程建设，重点抓好72个、总投资 1829.42亿元的省重点工程。鼓励社会资本参与重大工程建设运营，加快战略装车点、低热值煤电厂、外送电通道、新能源、新区两山防洪等重大项目建设，着力优化投资结构。积极谋划推动一批市场前景好、技术含量高、带动能力强的重大产业项目落地建设，提升产业竞争力，增添发展新动能。完善项目推进考核机制，建立重大项目储备、并联会审、联席会议和市县领导包联制度，及时解决资金、土地等突出问题，全力推动项目顺利实施。

二、统筹城乡基础设施建设，努力提升城镇化水平

深入贯彻落实中央和全省城市工作会议精神，坚持以人为本，创新城市规划、建设和管理理念，加强城乡居民共享的各类基础设施建设，提高城市宜居度，统筹城乡、区域协调发展。

强化规划引领。坚持多规合一，实现各类规划的衔接融合。市区要严格执行2015年出台的控制性详细规划，各县（市、区）要加快编制完善控制性详细规划和专项规划。进一步加强规划执行和监督执法工作，依法合规推进城镇化进程。

提升中心城市功能。统筹推进新城区、主城区一体化发展，抓住重点项目建设不放松，干好今年的、规划明年的、设想后年的，梯次推进、有的放矢，努力实现城市建设持续健康发展。初步搭就新区基本框架。把回迁安置作为当前新区建设的头等大事，以15个安置区建设为重点，完成绿化、道路硬化等配套工程，确保2万拆迁群众回迁入住。集中力量建设完善新区基本生产生活设施，加快推进市中心医院、儿童福利院、便民服务中心等新区10大工程建设，建成投用东属巴小学、大武小学，基本建成大武医院，确保回迁群众搬得进、住得稳。规划建设一批新区学校，支持太原铁路局建设车务段、工务段等相关项目，引进一批科技型大企业落户新区，提升新区吸纳人口、聚集产业功能。着眼长远发展，充分考虑与火车站餐饮住宿、商贸物流等站前经济的衔接融合，抓紧完善吕梁客运枢纽工程前期工作，力争标志性建筑新区长途汽车站年内开工；加快推进新区9座桥梁配套设施完善；力争开工建设方山麻地会—离石交口总长60公里的国道209线市区段改线工程，同时建设13.8公里新安大道（原国道209市区新城段）地下综合管廊；基本建成11.5公里“一河两带三湖”景观带，初步搭就新区发展框架。加快主城区提质改造。抓住国家棚户区改造的政策契机，以市区209国道沿线、滨河南北路“一线两路”为实施重点，在离石城区五个街道分别选取试点区域，统一政策标准，市区两级联动，启动实施一批城中村改造项目，探索路径，积累经验，逐步改变“城在村中、村在城中”及设施不全、出行不便、绿地不多、人口拥挤等落后局面。切实加大市区违法建筑、危旧建筑的拆迁力度，采取拆违还绿、改建扩绿、空地绿化、见缝插绿等措施，从城市主干道两侧、十字路口建筑入手，机关单位带头，成熟一片拆除一片，腾出更多空间建设绿地广场，向外疏解人口，在降低人口密度的同时，尽快提升城市品位。继续推进龙山、凤山、虎山三山绿化延伸，加快凤山公园提升改造等重点工程建设步伐，启动上山车道步道、公园绿地、休闲广场等项目前期规划设计。加快推进呈祥路和滨河东路南延、城北城南农贸市场、碧水桥、人行天桥和马茂庄大桥、世纪广场人防、高速东口景观道路提升改造以及龙凤南大街雨污分流等10大重点工程。继续抓好主城区集中供热、供气、供水、亮化、美化等市政维护和环境改善。同时，要建立城市建设绿色通道、责任倒查及重大事项24小时直通车制度，尽快形成一套严密高效的城市建设管理运行机制。

加强城市管理。宜居城市三分在建，七分靠管。要进一步厘清市区管理体制，明确和落实管理职责，全面加强城区拆迁、交通、环卫等队伍建设。在全市范围内开展城市管理综合整治活动，进一步规范建筑市场秩序，加快数字化城管步伐，扎实推进城市小区物业规范化管理。完成市区公交运营体制改革，组建国有企业控股的公交公司，淘汰51辆老旧黄标车，新增100辆新能源电动公交车，不断提升城市公共交通服务水平。要以园林城市建设为抓手，推进汾阳、岚县创建国家级园林城市，柳林创建省级园林县城工作，力争市区绿化覆盖率达到38%。要以卫生城市建设为抓手，启动汾阳、交城创建国家级卫生城市工作，持续引深城市环境卫生整治活动，创建3条省级容貌示范街道和3条保洁示范街道。要以智慧城市和海绵城市建设为抓手，全面加强城市地下管网综合管理，全面启动地下管线综合管廊建设规划编制，市区和孝义、汾阳先期组织实施；新建和改造城市道路130公里、各类市政管网505公里。改善人居环境。抓住国家棚户区和城中村改造政策机遇，年内实施棚户区改造1.2万户和城中村改造6777套。同步推进公共租赁住房建设，完善相关配套设施，抓好竣工项目的分配入住。启动8个县（市、区）、25个乡镇、70个自然村、13398户、38600人的采煤沉陷区治理搬迁工程；完成18910户农村危房改造任务。结合小城镇建设、生态旅游扶贫、古村落保护等，积极实施美丽宜居示范村建设，着力改善农村人居环境。要层层明确、下压、落实拆迁改造责任，推行“四个一”工作机制，做到每个项目一名县级领导挂帅，一个工作班子全程服务，一张工作推进“路线图”和“时间表”，一套考核奖惩办法，推动拆迁改造工作顺利实施。

加快交通建设。重点推进总投资367.8亿元、总长293.5公里的3条高速公路，总投资116.7亿元、总长228公里的5段国省道改造（改线）和投资6.5亿元、总长600公里农村公路改造建设，加快形成城乡公路互联互通的交通网络格局。全力推动兴县—静乐、离石—祁县高速公路项目年内开工，加快推进汾阳—石楼高速公路前期工作；确保新国道太克线一期工程完工，二期工程完成路基、桥隧工程，新国道孝辛线石口—石楼段升级改造工程上半年开工建设；力争国道209市区段、国道209省道340中阳城区段、国道307柳林城区段改线工程开工建设；重点新建改建县乡公路148公里，建设窄路拓宽工程324公里，建设通村硬化路128公里，改造建设一批旅游公路。

完善水利设施。推进中部引黄工程建设，加快惠及兴县、临县、石楼等9个县（市、区）、覆盖276万人口的小水网配套工程建设；力争龙门供水工程全线通水，离石千年水库竣工验收；做好碛口水利枢纽工程前期工作，争取列入国家“十三五”规划；推进峪口沟水库和规划内小水库前期工作，大力加强农田水利、农村饮水安全、防汛抗旱等工作。加快实施小流域综合治理、坝滩联治、坡耕地整治等重点水保生态治理，完成水土流失治理面积43万亩，实施生态修复22万亩。

推进电网建设。加快特高压输电网建设，确保榆横—潍坊吕梁段等项目按期完工。开工建设汾阳杨家庄等4项220千伏送出工程和文水胡兰、营口110千伏增容工程，实施柳林孟门等3个110千伏输变电工程，投运孝义迎宾等9个输变电工程。实施农网改造升级工程，力争全面消除“低电压”用户。

三、坚持改革开放，不断激发经济增长的动力和活力

树立问题导向，向改革要动力，向创新要活力，向开放要空间，着力引深“三大突破”，促进高新科技、现代金融与民营经济融合发展。

以电力体制综合改革为突破，全面深化重点领域改革。制定实施吕梁转型综改“十三五”规划和2016年行动计划，抓好转型综改试验区建设。推进电力体制综合改革，抓住我省列入国家电力体制改革试点机遇，加强电力统筹规划，合理优化电源和电网布局。充分利用我市日益完备的电网和资源、能源优势，推动现代载能企业自备电厂建设，同时有效扩大电力直接交易电量，加快推进更多企业通过电力交易平台实现直购电；推进电价市场化改革，有序放开输配以外的竞争性环节电价，分步实现电力市场化定价；发挥超低电价优势，扩大煤铝等资源产业的市场竞争力，增强对材料加工、大数据等高载能产业的吸引力，助推全市资源优势转化为经济优势，促进产业结构转型升级。深化财税体制改革。加大一般公共预算和政府性基金预算统筹力度，建立健全财政存量资金和预算安排统筹结合机制、库款余额与转移支付挂钩机制，最大限度盘活存量资金。统筹推进城镇户籍制度、商事制度以及林业、水利、供销合作社等各项改革。

以“三大突破”融合发展为抓手，提升市域经济整体实力。进一步总结推广孝义鹏飞、岚县继亨铸造等民营企业的经验做法，充分发挥我市资源优势，加强与上市公司资本、科技、管理等深度融合，在资源利用、资本合作、科技改造以及民营企业体制机制上进行大胆革新，提高企业核心竞争力，推动资源型经济转型发展。释放民营经济活力。认真落实我市加快民营经济发展的28条措施，全年新创企业2500户、新发展壮大规模企业22户以上；重点抓好100名民营企业优秀经营者高端培训和1000名有发展潜力的民营企业主创业能力提升培训，积极引导建立现代企业制度；支持社会资本建设中小微企业创业基地，新建市级创业基地2个。发挥科技创新支撑能力。充分运用电改成果，发挥梯度电价的优势，加速培育云计算产业。抓住华为公司在全国战略布局的机遇，引进华为云计算产业，建设立足山西、辐射华北地区的大数据中心，运用大数据改造传统产业，同时吸引一批行业领军企业及高精尖项目落户吕梁；继续加强与国防科大合作，加快天河云产业基地建设，推进柳林李家湾光电子等产业园区建设。落实国家创新驱动扶持政策，强化各级领导干部科技创新意识和企业创新主体地位，加强高新技术企业培育，支持创新型企业发展。创新人才体制机制，瞄准“高精尖缺”培育引进各类专业技术人才。增强金融保障能力。加快投融资体制改革，大力推广政府与社会资本投资合作（ PPP ）建设模式。加强与国开行、农发行等政策性银行的实质性合作，争取国家专项建设基金支持重大市政基础设施和公共服务设施建设。逐步完善融资担保体系，促进融资担保机构健康发展。深入推进农村信用社改制农商行，孝义年内完成改制，发挥好地方金融支持当地经济的积极作用。引导各类金融机构创新小额贷款产品，加大对扶贫开发的支持。鼓励企业直接上市融资，力争3户中小企业登陆“新三板”。引导本地企业采取多种方式与上市公司合作,鼓励金融机构降低贷款利率、优化融资结构、延长贷款期限，在解决企业困难的同时，化解金融风险。积极防范和重拳打击非法集资行为，全面优化金融生态环境。

以五大招商引资活动为重点，扩大对外开放步伐。进一步加强与先进发达地区交流合作，积极筹备召开电力体制改革暨高载能企业创新论坛，资源型企业与上市公司对接洽谈，旅游专题推介，“迎老乡、回故乡、建家乡”恳谈，互联网助推脱贫攻坚论坛等五大招商引资活动，力争全年招商引资签约额突破2500亿元。积极参与“一带一路”建设，主动融入京津冀、环渤海经济圈，推进红枣、核桃、小杂粮、铸造、汽车配件等外贸出口基地建设，促进优势产能和优秀企业“走出去”，吸引知名大集团、大企业进驻吕梁，努力提高我市经济外向度。

四、努力增加民生普惠成果，进一步提高共建能力和共享水平

以民之所望为施政所向，有效加强公共服务供给，切实保障群众权益，加快共享发展、和谐发展步伐。

推动教育优先发展。加大市区教育资源整合力度，盘活市区部分闲置资源合理布局中小学，努力满足城区人口增长对教育发展的需求。启动全市教师编制复核，整合农村空壳校教师编制保障城区学校。整合吕梁教育学院、离石师范、汾阳师范等教育资源，加快筹建吕梁高等幼儿师范专科学校，并在新区划拨建设用地启动校区建设，同时积极申请年内试招生。科学规划布局各类中小学校，启动新区中小学、幼儿园新改扩建工程，在市区规划建设2所公办幼儿园，吸引社会资本在城区发展一批普惠性民办中小学和幼儿园，缓解“入学难”“入园贵”的问题。改造农村幼儿园26所，继续实施全市52所城区中小学、幼儿园旱厕改造工程，推动交口、柳林、中阳等6县通过国家义务教育均衡发展评估认定。全力推动县级职教中心标准化建设，办好特殊教育。推进招生考试制度改革，落实乡村教师支持计划，加强师德师风建设，鼓励乡村教师扎根农村，推动教育内涵发展。

提高医疗卫生服务水平。市人民医院门诊楼年内完工，市中医院尽快恢复开诊，力争完成市中心医院、市疾控中心、市妇幼计生服务中心等项目主体工程，开工建设市护理职业学院。认真落实贫困人口医疗救助“9条”政策措施，重点解决贫困群众看病难问题。完善基层医疗卫生服务体系，推进分级诊疗，鼓励社会办医，强化乡村卫生院（室）建设；大力实施医疗卫生人才培养引进计划，重点加强儿科、老年医学、急救、康复和护理等方面的医技人才队伍建设。巩固县级公立医院综合改革成果，加快推进城市公立医院改革，引深基层医疗卫生机构综合改革。持续深入开展爱国卫生清洁运动，规范乡村垃圾处理清运，完成3.96万座农村卫生厕所改建任务。坚持计划生育基本国策，全面落实一对夫妇可生育两个孩子政策，促进人口均衡发展。严格落实属地监管责任，强化食品药品生产流通全过程日常检查，切实保障“舌尖上的安全”。

繁荣发展文化事业和文化产业。加快市图书馆、群众艺术馆建设，启动市体育馆、市体育场维修改造工程。加快推动县级“三馆一院”和基层综合性文化服务中心建设。广泛开展群众性文化体育活动，倡导全民阅读形成氛围，推出更多地方特色的文化精品力作。深入推进“乡村文化记忆工程”，加强非物质文化遗产保护。加快文化产业与体育、科技、旅游等融合发展，推动文化创意产业发展。

完善社会保障体系。稳步推进机关事业单位养老保险制度改革，提高退休人员基本养老金待遇水平。建立统一的城乡居民基本医疗保险制度，城乡居民医保财政补助标准由每人每年380元提高到420元。加快推进和完善大病保险和重特大疾病保障机制。全面开展重特大疾病医疗救助。继续提高城乡低保和农村五保保障标准，推动农村低保保障标准与国家扶贫标准相衔接。大力支持残疾人和慈善事业发展，鼓励支持民办养老机构建设，开工建设吕梁特仪工程。提高机关事业单位基本工资标准，落实县以下机关公务员职务与职级并行制度和乡镇工作补贴。严格落实最低工资标准，发布年度企业工资指导线和劳动力市场工资指导价位，推动实现居民收入增长和经济增长同步。对城镇失业人员、残疾人等就业困难群体实行精准帮扶、托底安置，确保年内城镇新增就业3.1万人。加强和创新社会治理。扎实推进“平安吕梁”建设，加快完善立体化社会治安防控体系。创新城乡基层自治和社区治理，加强基层综合服务管理平台建设和应用。加快社会诚信建设，推动形成守信激励、失信惩戒的制度机制和社会风尚。关爱农村留守儿童、妇女和老人，加强未成年人社会保护。加强和改进信访和调解工作，积极预防和化解社会矛盾，防止群体性事件发生。强化公共安全监管，继续保持打黑除恶高压态势，提高应急管理和防灾减灾能力，有效防范和处置突发性事件，切实保障人民群众生命财产安全。

重视国防动员、国防教育、人防和兵役工作。加强文物保护、抗震设防、民族宗教、档案方志工作。支持工会、团委和妇联等人民团体工作。做好统计、老龄、对台、气象、外事侨务、社团组织等工作。

各位代表，越是经济运行困难时期，我们越要更加关注民生、积极改善民生、着力惠及民生，让全市人民生活越来越好、幸福指数越来越高！

五、全面加强生态环境建设，加快推进绿色发展

坚持保护与开发并重，大力推动生态修复和环境治理，实现资源节约高效利用，满足人民群众对良好生态环境的新期待。

狠抓环境综合治理。持续落实大气、水、土壤污染防治措施，落实大气和水污染防治2016年行动计划，推进多污染物综合防治。持续推进区域面源污染整治、黄标车及老旧车淘汰、燃煤小锅炉整治、扬尘防治等重点行业污染整治工作。加快重点工业企业水处理环保设施提质改造，开展城市黑臭水体综合治理，保障集中式饮用水水源安全。加快岚县二期、中阳扩容等5个污水处理厂和市第二生活污水厂建设，确保年内建成投运。继续引深环境保护综合整治，严格落实网格化环境监管长效机制，依法严厉打击环境违法犯罪行为，持续改善环境质量。

促进资源节约和循环利用。落实能源和水资源消耗、建设用地等总量和强度双控要求，强化节能约束，加快文水振兴化工氯化铵节能技改（二期）等18个项目节能技术改造，重点推进汾阳国峰粉煤灰利用等10个资源综合利用和清洁生产项目。大力推动绿色建筑发展。

推动生态保护和修复。重点抓好吕梁山生态脆弱区林业建设，完成造林57.34万亩。继续实施市区建成区绿化延伸工程，加强3.8万亩三山绿化林木的抚育管理。大力推进矿山生态环境治理和地质灾害防治，加强水土保持监督执法。积极倡导绿色发展、绿色消费理念，让吕梁天蓝水净、地绿山青！

六、高度重视安全生产，持续推动安全生产形势明显好转

安全生产高于一切，安全责任重于泰山。我们必须始终牢记“三个绝不能过高估计”的基本判断，始终坚持敬畏生命、敬畏责任、敬畏制度，时刻绷紧安全生产这根弦，做到安全意识不松、投入标准不降、监管力度不减。

严格落实安全生产责任。按照“党政同责、一岗双责、齐抓共管”和“三必管”要求，进一步完善安全生产责任体系，落实各级政府、职能部门安全生产监管责任和企业安全生产主体责任。严格执行企业安全生产费用提取和使用管理规定，建立安全生产投入长效机制，不断改善安全生产基础设施和条件。

深化隐患排查治理。积极推广“政府购买服务、专家会诊指导、企业排查治理、部门执法监督”的隐患排查治理机制，深入开展煤矿、非煤矿山和尾矿库、道路交通、危险化学品、油气输送管道、特种设备、消防以及农产品质量安全等重点行业领域的安全生产大检查。严格按照“四个一律”要求，坚决打击非法违法生产经营建设行为。

加强依法治安和基层基础工作。加大依法治安力度，全面推进安全标准化建设，大力推广新工艺、新装备，不断提高本质安全生产水平。进一步强化安全生产基层基础，加强安全应急救援体系和能力建设，坚决杜绝重大事故，遏制较大事故，减少一般性事故，促进全市安全生产形势持续稳定好转。

七、以增加农民收入为核心，扎实抓好脱贫攻坚和“三农”工作

坚持把脱贫攻坚作为经济社会发展的主战场，作为首要的政治任务和第一民生工程，以脱贫攻坚统揽经济社会发展，坚持产业扶持、技能扶持、资本扶持和不断改善基本生产生活条件“3+1”的精准扶贫路径，按照习近平总书记“六个精准”“五个一批”脱贫攻坚要求，围绕实现“两不愁、三保障”目标，精准实施“三个一”扶贫行动、“十大重点工程”，不断加快脱贫致富进程，确保全年270个以上贫困村摘帽、10万以上贫困人口脱贫。全面开展“三个一”扶贫行动计划，扶持全市20%以上贫困人口精准增收。吕梁山片区“三个一”扶贫行动计划，是省委、省政府以吕梁为主体量身定做的一项增收脱贫政策，我们要抓住机遇，全力推进，力争年内11.4万贫困人口受益。围绕增加贫困人口经营性收入，全面实施经济林提质增效工程。投资3.05亿元，在兴县、临县、石楼等12个县（市、区）新发展核桃林20万亩、品种改良6万亩，完成红枣、核桃综合管护20万亩，坚持新栽项目高起点建设、综合管护专业化施工、品种改良优质化实施，确保覆盖181个贫困村4.9万贫困人口。围绕增加贫困人口财产性收入，大力实施光伏扶贫工程。启动实施6个国定贫困县光伏扶贫规划，尽快组建扶贫开发投资公司，与晋能集团合作，按照“五统一、两集中”建设模式，采取村级分布式、村级集中式、户用分布式和大型地面站建设四种方式，试点先行方山25兆瓦、覆盖1.3万无劳动能力贫困人口的光伏电站，同步推进临县、石楼等5个国定贫困县282兆瓦、覆盖13.2万无劳动能力贫困人口的光伏电站建设，力争年内6万贫困人口实现资产性收益全覆盖。围绕增加贫困人口工资性收入，积极实施以护工护理为重点的培训就业工程。以市卫校等5所学校为

“吕梁山护工”培训基地，带动各类社会组织积极参与，全面推进以护工护理和家政服务为重点的1万名贫困人口培训工程；加快完善定向培训、订单就业、跟踪服务等培训就业体系，力争一半以上培训结业人员稳定就业，人均月收入达到4000元以上。

精准实施脱贫攻坚“十大重点工程”，确保对所有贫困人口实行精准扶持。加快易地扶贫搬迁工程，抓住棚户区改造、采煤沉陷区治理、房地产去库存等政策机遇，按照五年任务三年完成的要求，统一规划、分步实施，通过行政村就近集中安置、建设移民新村集中安置等六种方式，年内完成2.05万贫困人口的易地搬迁任务，吸纳更多贫困人口就业创业。深入推进“8+2”农业产业化，坚持提档扩面、精准到户、多元互补，重点打造交口食用菌、兴县杂粮、岚县马铃薯、方山中药材等基地县；同时，以平川四县和离石区为重点，大力发展设施蔬菜产业，积极发展特色养殖产业。大力实施企业产业扶贫工程，积极推广离石泰化集团、中阳厚通养殖公司、石楼富民林牧公司等企业参与产业扶贫的典型经验，认真总结推广“借羊还羊（草）”扶贫模式，发动全市各类企业尤其是资源型企业和规模以上企业，通过资源开发、产业培育、市场开拓以及“公司+基地+农户”等多种形式，带动贫困乡村实施区域化、规模化产业开发；坚持把产业扶贫作为当前重要的发展机遇，注重规划和推出一批扶贫项目，引导支持社会资本进入贫困地区，引进一批大企业参与产业扶贫，努力实现农企双赢。高度重视教育和医疗扶贫，年内市财政安排2354万元，对所有贫困县农村中小学寄宿生提供营养餐补助，对所有贫困家庭高中生免除学杂费，对考入专科以上贫困家庭大学生给予一次性学费资助；安排918万元，全面提高贫困人口新农合门诊统筹补偿封顶线，将贫困人口在县、乡医疗机构新农合补偿比例提高到80%和90%，免除五保对象在市内新农合定点医疗机构万元以内的就诊费，提高全市贫困人口新农合个人缴费政府补助比例。扎实推进生态扶贫工程，要认真总结和推广岚县“购买式造林”、石楼“沟域治理”“龙头企业带动”等扶贫模式，加快发展沟域经济，打造一批绿色生态、一二三产业融合、特色鲜明的沟域经济产业；支持贫困群众通过以工代赈、造林出让等形式参与生态治理，积极推进25度以上坡耕地退耕还林还草。积极实施基础设施和公共服务改善工程，力争让更多贫困群众普遍住上安全房、喝上干净水、用上卫生厕所、走上平坦路，逐步改善贫困群众生产生活条件。同时，落实好相关职能部门的扶贫责任，精准实施金融扶贫、电商扶贫和乡村旅游扶贫等各项重点扶贫工程，积极引导社会力量广泛参与脱贫攻坚，最大限度地整合各类资源，精准发力，带动贫困群众脱贫致富奔小康。

统筹发挥包村领导、驻村工作队和第一书记三支扶贫力量的管道作用，确保各项脱贫政策措施和项目资金精准到村到户。要充分发挥考核的指挥棒作用，不断创新脱贫攻坚考核机制，建立健全精准投入机制，严格落实“一把手”负总责的脱贫攻坚领导责任制，加快形成专项扶贫、行业扶贫、社会扶贫“三位一体”的大扶贫格局。总结推广石楼县“54321”领导干部精准帮扶机制，严格落实领导干部结对帮扶等制度措施，让所有包村领导、驻村工作队和第一书记真正沉下去、联系紧、扶到位。要全面实施记录脱贫，由帮扶对象和贫困户共同记载精准脱贫的措施落实、资金到位、项目进展、问题成效等，并及时录入永久性电子数据平台，确保精准脱贫全程留痕。要坚持日记录、周报告、月总结，加快建立“市有库、县有档、乡有账、村有册、户有簿”的精准脱贫大数据档案，为鉴定贫困户脱贫出列和考核帮扶对象，提供全面、准确、详实的大数据，实现对全市脱贫攻坚的动态管理、科学研判和客观评价。

始终把“三农”工作作为重中之重，持续增加农民收入。全面落实各项强农惠农富农政策，大力推进农业生产规模化、集约化、标准化发展。实施藏粮于地、藏粮于技战略，开展粮食高产创建和新技术示范展示，稳定粮食生产面积。强化农业科技创新，加强与省农科院等科技院所合作，力争在红枣防裂果以及核桃抗晚霜冻等方面取得新突破。全面启动全国农业标准化示范市创建工作，围绕设施蔬菜、马铃薯、小杂粮、食用菌等特色产业，重点发展无公害、绿色、有机产品，力争年内建成标准化示范区30个、面积50万亩、标准化养殖场15个。加强新型职业农民培训，培育一批职业农民电商、职业菜农、职业果农、职业牧农；抓住我市集中实施低热值煤电厂、铝工业和铁路战略装车点、高速公路、大水网等重大项目建设机遇，重点围绕增加农民的工资性和经营性收入，引导培训一批农村劳动力在运输、装载、餐饮、家政等劳动密集型岗位就业，实现稳定增收。加强产销对接、农超对接，引导支持农民合作社在城郊或重点城镇建立农产品直销店，注重发挥电商平台作用，有效拓宽农产品销售渠道。加快农村综合改革，基本完成农村土地承包经营权确权登记颁证工作，加强农村集体“三资”管理，建立农村产权流转交易市场。大力培育新型农业经营主体，创新农村土地流转和农村规模经营方式。做好第三次全国农业普查工作。

各位代表，脱贫攻坚任务艰巨、责任重大，我们要抓住用好国家各类各项政策措施,举全市之力,集全民之智,全面打响这场硬仗，坚决保证“十三五”脱贫攻坚初战有效、首战告捷！

八、坚持务实进取，切实加强政府自身建设

面对艰巨繁重的发展任务，我们必须加快转变政府职能，持续改进工作作风，全面提升政府的执行力和公信力。

加强廉政建设。严格落实党风廉政建设责任制和主体责任，认真履行“一岗双责”，把廉洁从政贯穿到政府工作各个环节。严格执行中央“八项规定”，强化对公共资金、公共资源、国有资产的行政监察和审计监督，严厉查处违纪违法案件，始终保持惩治腐败、狠刹“四风”高压态势。稳妥推进公务用车制度改革。完善厉行节约长效机制，强化“三公”经费管理，压缩一般性支出，集中有限财力优先保工资、保运转、保民生。

严格依法行政。落实国家法治政府建设实施纲要，自觉运用法治思维和法治方式履行政府职能，把政府各项工作纳入法治轨道。完善“两清单”“两平台”运行制度，推动“六权治本”向乡村两级延伸。健全重大行政决策机制，提高政府决策的科学化、民主化水平。深化行政审批制度改革，加强事中事后监管，持续推进简政放权、放管结合、优化服务。自觉接受人大法律监督、工作监督和政协民主监督，全面接受人民监督。大力推行政务公开，推广电子政务和网上办事，让企业和群众办事更加便捷，营造宽松发展环境。

狠抓工作落实。深入践行“三严三实”，认真开展“两学一做”学习教育，牢固树立政治意识、大局意识、核心意识、看齐意识。建立重点工作督查评估和行政问责机制，坚决纠正不作为、乱作为，坚决克服懒政、怠政，坚决惩处失职、渎职，着力解决缺位、越位、不到位的问题。坚持以上率下，从市政府班子做起，带头包贫困村、帮扶困难企业、包联重点项目，始终保持积极进取的精神状态，引导和带动政府系统各个层面崇尚实干、敢于担当，让夙兴夜寐、激情工作成为政府系统运转常态，用全体政府工作人员的辛苦指数换取全市人民的幸福指数。

各位代表，吕梁发展中的困难需要我们克服，吕梁人民对美好生活的向往激励我们奋进，吕梁新的发展篇章等待我们书写。让我们在省委、省政府和市委的坚强领导下，凝心聚力、只争朝夕，夙夜在公、勤勉工作，为加快实现“新布局、新发展、新形象”而努力奋斗！
